# Impact of a transition nurse program on the prevention of thirty-day hospital readmissions of elderly patients discharged from short-stay units: study protocol of the PROUST stepped-wedge cluster randomised trial

**DOI:** 10.1186/s12877-016-0233-2

**Published:** 2016-03-03

**Authors:** Pauline Occelli, Sandrine Touzet, Muriel Rabilloud, Christell Ganne, Stéphanie Poupon Bourdy, Béatrice Galamand, Matthieu Debray, André Dartiguepeyrou, Michel Chuzeville, Brigitte Comte, Basile Turkie, Magali Tardy, Jean-Stéphane Luiggi, Thierry Jacquet-Francillon, Thomas Gilbert, Marc Bonnefoy

**Affiliations:** Hospices Civils de Lyon, Unité de recherche sur la qualité et la sécurité des soins du Pôle Information Médicale Evaluation Recherche , Lyon, 69003 France; Hospices Civils de Lyon, Service de Biostatistique, Lyon, 69003 France; Université de Lyon, Lyon, 69000 France; Université Lyon 1, Villeurbanne, 69100 France; CNRS, UMR 5558, Laboratoire de Biométrie et Biologie Evolutive, Equipe Biostatistique-Santé, Villeurbanne, 69100 France; Hospices Civils de Lyon, Centre Hospitalier de Lyon Sud - Pavillon Michel PERRET, Pierre-Bénite, 69495 France; Centre Hospitalier Général d’Annecy, Pringy, 74374 France; Centre Hospitalier Alpes Léman, Contamine Sur Arve, 74130 France; Hôpital Édouard Herriot - Pavillon E, Lyon, 69003 France; Clinique des Portes du Sud, Vénissieux, 69200 France; Centre Hospitalier de Saint-Chamond, Saint-Chamond, 42400 France; Centre Hospitalier Gériatrique du Mont d’Or, Albigny-sur-Saône, 69250 France; CH Bourg en Bresse, Bourg-en-Bresse, 01012 France

**Keywords:** Care transition, Patient readmission, Stepped-wedge design, Elderly

## Abstract

**Background:**

In France, for patients aged 75 or older, it has been estimated that the hospital readmission rate within 30 days is 14 %, a quarter being avoidable. Some evidence suggests that interventions “bridging” the transition from hospital to home and involving a designated professional (usually nurses) are the most effective in reducing the risk of readmission, but the level of evidence of current studies is low. Our study aims to assess the impact of a care transition program from hospital to home for elderly admitted to short-stay units.

**Methods:**

This is a multicentre, stepped-wedge cluster randomised trial.

The program will be implemented at three times of the transition: 1) during the patient’s stay in hospital: development of a discharge plan, creation of a transitional care file, and notification of the primary care physician about inpatient care and hospital discharge by the transition nurse; 2) on the day of discharge: meeting between the transition nurse and the patient to review the follow-up recommendations; and 3) for 4 weeks after discharge: follow-up by the transition nurse.

The primary outcome is the 30-day unscheduled hospital readmission or emergency visit rate after the index hospital discharge.

The patients enrolled will be aged 75 or older, hospitalized in an acute care geriatric unit, and at risk of hospital readmission or an emergency visit after returning home.

In all, 630 patients will be included over a 14-month period. Data analysis will be blinded to allocation, but due to the nature of the intervention, physicians and patients will not be blinded.

**Discussion:**

Our study makes it possible to evaluate the specific effect of a bridging intervention involving a designated professional intervening before, during, and after hospital discharge.

The strengths of the study design are methodological and practical. It permits the estimation of the intervention effect using between- and within-cluster comparisons; the study of the fluctuations in unscheduled hospital readmission or emergency visit rates; the participation of all clusters in the intervention condition; the implementation of the intervention in each cluster successively.

**Trial Registration:**

This study has been registered as a cRCT at clinicaltrials.gov (identifier: NCT02421133). Registered 9 March 2015.

## Background

In France in 2010, 33 % of subjects aged 75 years or older were hospitalised at least once in a short-stay care unit [1]. The rate of unscheduled hospital readmissions within 30 days has been reported to reach 14 % for such patients (IC 95 % [12,0–16,7]), with nearly one quarter of these readmissions being preventable [2, 3]. There are numerous reasons for these readmissions: those linked to the patient (comorbidities, polypharmacy, functional disabilities, malnutrition, and musculoskeletal, psychocognitive and sensory disorders), to his environment (social isolation, absence of a care network, unsuitable home), and to the health care system (care during the hospital stay, hospital-to-home transition including management of patient discharge, and patient follow-up after discharge) [4].

Several literature reviews have attempted to identify effective interventions for managing the hospital-to-home transition [5–8]. Such interventions “aim, during and after hospitalisation, to prevent a breakdown in the continuity of care and to reduce the occurrence of adverse health effects, including preventable readmissions [5].” It was concluded that, in order to be effective, such interventions must combine several actions at three steps of the transition: 1) before the patient leaves the hospital, 2) at the time of discharge, and 3) within 48 h and up to 30 days after discharge. A decrease in readmissions within 30 days is the most frequently used criterion for evaluating the quality of in-hospital care and of the hospital-to-home transition. The implementation of an intervention program requires the identification of patients at risk of hospital readmission. Finally, it must be possible to adapt the intervention to each situation according to the specific needs of the patient and those supporting him.

Three-step transition interventions (i.e., bridging interventions) involving a specially assigned professional appear to be the most effective in reducing the risk of hospital readmission, but the level of evidence of current studies remains weak [5–7]: there have been few randomised controlled trials or multicentre studies, and the tested interventions have been heterogeneous in terms of the choice of the transition professional (generally nurses), the definition of the population at risk for readmission that is targeted by the intervention, and the follow-up time after hospitalisation [7]. In addition, the existing data do not permit the assessment of medico-economic impact [9].

### Objective

This study aims to assess the impact of a care transition program from hospital to home on the 30-day unscheduled hospital readmission or emergency visit rate after the index hospital discharge, for people aged 75 years or older who are admitted to short-stay care units.

The other objectives are to evaluate the impact of this transition program on the safety and quality of home-based care (mortality, quality of life, and patient satisfaction); to evaluate the implementation of the program (time required to set up the help scheme, and timing and frequency of communication among the various actors); and to carry out a medico-economic assessment including a cost-effectiveness analysis at 30 days and a budgetary impact study at 12 months.

## Methods/design

### Setting

The study is to take place in nine short-stay geriatrics units in hospitals across the Rhône-Alpes region of France (two university teaching hospitals, one private hospital, and five public hospitals) over a period of 14 months.

### Study design

This quasi-experimental study is a multicentre prospective, stepped-wedge cluster randomised trial with a continuous recruitment, short exposure design (participants are exposed for a short period with single measurement at a fixed time after the start of their exposure) [10–12].

**Fig. 1:**
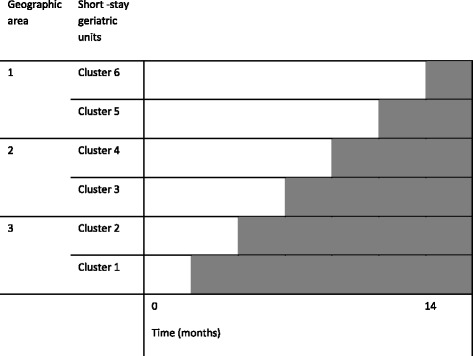
Stepped-wedge study with a continuous recruitment, short exposure design. Shaded areas indicate transition program exposure and unshaded areas indicate control exposure

The nine short-stay geriatric units will be grouped into six clusters (each cluster comprising one to two short stay units), taking into account their patient recruitment capacity and their geographic proximity. The six clusters will be divided into three geographic areas. The order of rollout will be randomised within each geographic area (see Fig. 1). There will be no randomisation between geographic areas.

At regular intervals, the clusters will cross over from control to intervention. Each cluster will therefore receive both control and intervention situations consecutively. Data will be collected in all clusters at each time step.

### Time steps

The study includes seven time steps (T0 to T6) with a 2-month period between steps. All clusters will start with the control situation (no care transition program) at the beginning of the study. At each time step, a new cluster will cross over from the control situation to the implementation situation. Each cluster will start the implementation of the program at a different time step. At T7, all clusters (and hence all hospitals) will have received the care transition program.

### Participants

The inclusion criteria are: patient hospitalised for 48 h or more in one of the short-stay geriatrics units participating in the study, aged 75 or older, living at home and with home as the planned place of discharge after admission, and at risk of hospital readmission or emergency visit after discharge. A patient will be considered to be at risk if he meets two or more of the following criteria (derived from the Triage Risk Screening Tool and from the 2013 French recommendations) [5, 13]:

- Problems in performing activities of daily living (assessed with the Index of Independence in Activities of Daily Living or ADL, and the Instrumental Activities of Daily Living or IADL), excluding the problem of urinary incontinence;
- One unscheduled hospital admission during the three previous months, or two or more unscheduled hospital admissions during the previous year;
- A geriatric syndrome such as: two or more falls during the 12 previous months, malnutrition (as defined by the French National Authority for Health), cognitive impairment (degenerative diseases, Alzheimer’s disease, or other dementia), or depression;
- One or more chronic diseases with high risk of acute decompensation or hospital readmission (i.e., chronic heart failure, chronic respiratory failure, etc.);
- Polypharmacy (defined as daily intake of five or more drugs); and
- An unfavourable social situation (social exclusion, weakness regarding the home helper).

The exclusion criteria are:

- Patient living in a retirement home (i.e., a nursing home or a residential home for the elderly);
- Patient hospitalised at home;
- Patient living at home, but at a distance of 30 km (18 miles) or more from the hospital of index admission; and
- Patient already included previously in the study (each patient participates in the trial only once).

### Intervention

The hospital-to-home care transition program will be implemented at three times: during the patient’s stay in hospital, on the day of discharge, and for 4 weeks after discharge.

*During the patient’s stay in hospital: development of the discharge plan and creation of the transitional care file, and notification of the primary care physician about inpatient care and hospital discharge*

- The medical team will proceed as usual to deliver a medical and geriatric assessment of the patient and to create a discharge care plan in accordance with the local common ways of care in the unit.
- The transition nurse (TN) will create a transitional care file including information about the patient (inpatient medical and nursing care plan, medications), the discharge plan, and the contact information of the relevant primary care providers.
- The TN will notify the patient’s primary care physician (by telephone, email, or fax) regarding the date of discharge to home, any potential medical problems, and the discharge care plan.
- If necessary, the TN will consider a home assessment visit before the patient is discharged from hospital in order to more accurately assess for aids and contribute to improving the discharge care plan.

*The day of hospital discharge (or shortly before discharge): meeting between the transition nurse and the patient to review the follow-up recommendations*

- The TN will check that medications are prescribed in accordance with the discharge plan, that the patient and his caregiver understand the prescription and its modifications, and that they are informed regarding planned appointments and biological monitoring.
- The TN will give a handover sheet (intended for the primary care providers) to the patient and his caregiver. This sheet, which will be owned by the patient, will facilitate cross-information transfer.
- The patient will be given the telephone number of the TN in case he has questions after returning home.

*For 4 weeks after hospital discharge: follow-up by telephone and home visit*

**Fig. 2:**
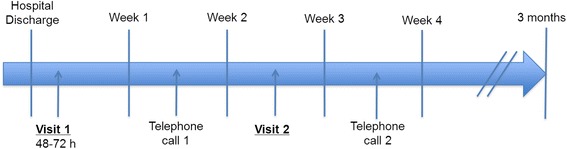
Follow-up within 4 weeks of hospital discharge

- The TN will follow up on the patient once a week for 4 weeks, through at least two home visits and two telephone calls (Fig. 2).

*Profile and role of the transition nurse*

The prerequisite for the TN is to have worked in geriatric units (in acute care or in rehabilitation care facilities). The TN will be trained by the research team in the components and tools of the intervention and in the organisation of primary care. In each participating hospital, the TN will meet the hospital care providers and will establish contact with the local primary care providers who usually take care of the patients at home. Table 1 describes what the TN will do for the intervention patients.

**Table 1:** Description of activities of the transition nurse (TN)

Time of the transition	Action of the TN	Main contact	Tool implemented
During the patient’s stay in hospital	Collect data about the patient, his caregiver, his primary care physician, and current primary care providers;	Medical team and social workers of the discharging hospital	Transitional care file intended for the TN: hospital part
	Verify that the admission geriatric assessment has been carried out by the medical team and complete it if necessary;		
	Develop the discharge plan in collaboration with the physicians, nurses, physiotherapists, and social workers of the hospital.		
When the day of hospital discharge is set	Check that the date of returning home is known by the patient, his caregiver, and the primary care physician;	The patient's primary care physician	
	Check that the discharge summary and plan have been transmitted to the primary care physician;		
	Check the organisation of the transport if needed;		
	Check that a primary care physician visit is planned (at home or in office) during the month following discharge;		
	Prepare the handover sheet, which includes the meetings scheduled (medical exams, biological monitoring), the contacts scheduled with the TN (by telephone or home visit), the telephone number and timetable where the TN can be reached, and the contact information of the primary care providers.		
The day of hospital discharge	Check that the prescriptions for the discharge care plan are written (medications, physical therapy, medical equipment, etc.);	Patient and his caregiver	Handover sheet intended for the patient and the primary care providers
	Explain the discharge plan to the patient or his caregiver;		
	Give the completed handover sheet to the patient or caregiver and verify that the visits scheduled are planned in accordance with the patient or caregiver's availability;		
	Check that the discharge plan will be implemented with the social worker;		
	Check that the inpatient nursing care plan, along with the medical discharge summary, is in the handover sheet.		
After hospital discharge: follow-up by home visit and telephone	Verify the effective implementation of human and material aid; ask about difficulties and seek to resolve problems;	Patient and his caregiver Primary care providers Geriatrician	Transitional care file intended for the TN: home part
	Help to prevent the risk of falls by having a look at the environment at home;		
	Ensure good medication compliance; verify the autonomy and clinical status of the patient, and contact stakeholders (nurse, primary care physician, hospital physician) if necessary;		
	Retrieve the results of biological monitoring and of medical visits; answer questions from the patient and his caregiver;		
	Provide regular reports to the primary care providers (by completing the handover sheet) and to the geriatrician.		

### Control period

No intervention liable to affect the care provided for the patients, the organisation of care, or the practices of health care professionals will be implemented during the control period (time steps without intervention). The patients will be discharged according to the usual care plan of each participating hospital. The medical team will proceed as usual to deliver a medical and geriatric assessment of the patients according to existing recommendations. The communication of information to the primary care providers (nurse, primary care physician, etc.) is left to the discretion of the medical teams of the discharging hospitals, according to their existing work habits.

In the control period, the normal care practices will be assessed following the Template for Intervention Description and Replication (TIDieR) guidelines [14].

### Outcomes and measurements

#### Primary outcome

The primary outcome is the 30-day unscheduled hospital readmission or emergency visit rate after the index hospital discharge. Unscheduled hospital readmissions are hospitalisations that are not planned at the time of discharge (for example, hospitalisation through Accidents and Emergency departments or upon the request of the primary care physician). A follow-up at 30 days after discharge evaluates care transition failures, before and after hospital discharge.

#### Secondary outcomes

The secondary outcomes are:

Patient safety and quality issues at home:

- Survival without hospitalisation after discharge (within 30 days)
- Mortality rate at 30 days after discharge
- Quality of life measured with the French version of the EuroQol EQ-5D-3 L [15] at 30 days after discharge
- Patients’ satisfaction with the quality of preparation for post-hospital care measured with the Care Transition Measure® questionnaire [16] at 30 days after discharge. The French version of this questionnaire will be validated separately.

Delivery of the intervention:

- Time period from the decision relative to the date of discharge to the discharge itself
- Time period from discharge to the implementation of human and material aids at home
- Time period for the communication of relevant information to the primary care providers
- Number of contacts between the TN and the primary care providers or the hospital providers after discharge

### Medico-economic study

The medico-economic study has a “piggy-back” design because it is added to the clinical study: the data necessary for the medico-economic study will be obtained from the detailed observation of the resources used for each patient included. Data collection will be done prospectively.

The medico-economic study will have two components.

- The first component will be a cost-effectiveness analysis of the main criterion assessed in the study. It will provide a cost per unscheduled hospitalisation or emergency visit prevented. The health insurance perspective is chosen. The time limit is 30 days after the index hospital discharge. The costs assessed will be direct medical expenses (hospital expenses, reimbursement of consultations or examinations, etc.), non-medical expenses (reimbursement of transport), and care transition program expenses (time spent by the TN for each patient at the three times of the transition).
- The second component will be a budgetary impact study of the implementation of the intervention. The time limit is 12 months after the index hospital discharge. Health insurance and societal perspectives will be considered. The costs assessed will be direct and indirect medical expenses (medical and social expenses, family expenses, institutional care expenses, etc.) and care transition program expenses. Various scenarios may be proposed in order to study the financial impact of the care transition program on the funders.

### Sample size calculation

The number of patients has been determined according to a compromise between the inclusion ability of the units and the needed power, using the method of M.A. Hussey and J.P. Hughes [17]. The following hypotheses were used:

- A 30-day unscheduled hospital readmission or emergency visit rate after discharge of 20 % in the control group [2].
- A 10 % rate in the intervention group: the absolute risk reduction of the 30-day unscheduled hospital readmission or emergency visit rate ranges from 2.0 to 28.1 points according to previous studies with a bridging intervention based on a transition coach [18–24].
- A variation coefficient between clusters of 10 %. In cluster randomized trials, the variation coefficient is generally between 0.1 and 0.4. We have chosen a value of 0.1, because the variation of the outcome between units is supposed to be low. Furthermore, M.A. Hussey and J.P. Hughes have shown that, because the stepped wedge design uses both within-cluster and between-cluster information, power is relatively insensitive to variations of the coefficient of variation [17].

Under these hypotheses, for 6 clusters, 7 time periods, and an unilateral alpha risk of 5 %, the inclusion of 14 patients per cluster and per time period allows to reach a power of 70 %. The whole number of patients is 588. To allow for about 10 % missing data on the primary outcome, 15 patients per cluster and per time period will be included, for a total number of 630 patients.

A unilateral test has been chosen as it seems unlikely that exposure to the care transition program will increase the rate of unscheduled hospital readmissions or emergency visits within 30 days: these events are the result of hospital care factors, primary care factors, and patient factors. It does not seem possible for the intervention tested in this study to intensify the effect of these factors. The literature reviews do not report a significant increase in readmission rates associated with interventions on the hospital-to-home transition for older patients [5–8].

### Blinding

Health care providers delivering the intervention, participants, and researchers will not be blinded to the intervention status. Contamination between clusters will nonetheless be prevented, because it is impossible to implement the care transition program without a TN. Primary outcome measures will be carried out by a clinical research associate who will contact each patient by telephone and who will not be blinded to the participant's status as intervention or control, for practical reasons. Information bias will be minimised, because the primary outcome (the 30-day unscheduled hospital readmission or emergency visit rate) is an objective criterion that does not require interpretation (contrary to clinical events). Data analysis will be blinded to allocation.

### Ethical approval and informed consent

Approval for the study was obtained from the hospital ethics committee, the Institutional Review Board, and the French Data Protection Authority (CNIL). Consent of the health care staff was sought prior to the study. The patients will be offered the opportunity to participate, and their consent (or, rather, non-refusal) will be written down in the medical file. As stipulated by French law, however, no signed patient consent will be required, given the study methodology and the type of intervention.

### Data analysis

Data analysis will be performed by the data management and analysis centre. The analyses will be carried out with the latest version of the R software environment. The lme4 package will be used to implement the generalised linear mixed-effects models. An intention-to-treat analysis will be performed at the cluster level, after the end of the last step (T7).

All of the characteristics collected will be subjected to a descriptive analysis. The comparability of the patients (between the intervention group and the control group, overall, at each time period and within each unit) will be verified.

The univariate analysis will consist in quantifying and testing the effect of the intervention on the proportion of patients undergoing an unscheduled rehospitalisation or an emergency visit.

First, the result criterion will be outlined within each unit and for each period (without the intervention, and then with the intervention). The average proportion will be compared between the two periods using a matched and weighted *t*-test in order to take into account the heterogeneous numbers of patients included in the various units.

As a second step, a mixed-effects logistic regression model with a random effect on the intercept will be used to model the probability of an unscheduled rehospitalisation or emergency visit taking into account the intra-cluster correlation. The effect of the intervention will be quantified using an odds ratio with a 95 % confidence interval. A random effect on the effect of the intervention can be added in order to quantify any heterogeneity of the impact among the units.

The possible evolution of the result criterion over time will be considered by integrating a period variable into the model, enabling the quantification of the effect of each time period with respect to a reference period (the first time period). In the event a tendency is highlighted, the period variable could be introduced into the model as an ordinal variable. This analysis will make it possible to quantify the effect of the intervention after adjusting for a possible time effect. Also, it will be possible to search for an interaction between time and the effect of the intervention.

The analysis can be adjusted on the patient characteristics that are likely to affect the result criterion.

### Time frame

The planned inclusion period is 14 months:

- Time period 1. During this 2-month period, no specific intervention will be implemented as part of the study.
- Time periods 2 through 7. The intervention is implemented step by step, every 2 months, in the six clusters. At the end of time period 7, all clusters will have received the intervention for two to 12 months depending on the order in which the different clusters receive the intervention.

### Trial status

Patient enrolment began the 1st of July 2015. Data are collecting.

## Discussion

### Discussion of the study design

A stepped-wedge trial presents methodological advantages [10, 11, 25, 26]. The clusters act as their own controls because they receive both the control and treatment conditions. Therefore, the intervention effect can be estimated from both between- and within-cluster comparisons. In addition, a stepped-wedge trial can take into account the changes in the main criterion over time (fluctuations in unscheduled hospital readmission or emergency visit rate) and enables the quantification of the effect of time on the effectiveness of the intervention (the learning effects).

This type of trial also presents benefits with regard to implementation [10, 11, 25, 26]. It allows the intervention to be administered successively in the participating centres, which can facilitate the organisation of a study in routine practice. The implementation of the intervention at regular time-step intervals also allows for improvement of the intervention or its delivery where necessary before the next implementation step, even if this may introduce differences in the intervention between clusters [10].

Indeed, learning effects may influence the effectiveness of the intervention [26]. First, the TN may become more experienced after enrolling each new cluster, which may lead to differences in the program (albeit small ones) between clusters. Subsequently, this may have an impact on the estimated intervention effect across clusters. Second, clusters will differ in the amount of time spent on the intervention. Eventually, clusters that switch at the first step will be more experienced with the intervention than clusters that switch at later steps. This may also influence the estimated intervention effect. Nonetheless, both types of learning effects can be modelled.

Finally, a stepped-wedge trial makes it possible for each participating centre to benefit from the intervention. This inclusiveness is a motivational factor for taking part in the study, and it also conforms to good ethical practice given that the intervention is expected to do more good than harm.

A trial that randomises patients is not relevant given the close collaboration between the TN and the hospital care team in the context of the care transition program. It is impossible to consider that the care team could alter its organisation based on the randomisation group of each patient. In such a case, the risk of contamination between intervention and control participants would be too high.

### Discussion of the intervention

The intervention tested herein may be referred to as a bridging intervention because it is carried out both before and after patients leave the hospital. However, this type of intervention is not precisely defined, in particular with respect to the duration of follow-up after hospitalisation and to its components. Indeed, the literature reviews [5–8] have pointed out the heterogeneous nature of the interventions tested (combining up to eight different actions), the populations studied, and the assessment criteria measured. They have also pointed out an inadequate description of how the intervention unfolds, and the absence of data regarding the context of the implementation that could explain the success or failure of the intervention in question.

Our program takes its inspiration from interventions using a TN that have shown a significant reduction in unscheduled hospital readmission and/or emergency visit rates in randomised controlled trials in geriatrics. Five studies identified in the literature reviews [6, 7] met the above criteria and were available when our protocol was developed [19, 20, 22, 24, 27].

In these five studies, the nurse was usually an advanced practice nurse [19, 20, 24, 27]. The nurse's intervention was centred on the hospitalised patient [20, 24], on health care professionals in and out of hospital [19, 22], or on both [27]. When the intervention targeted the patient, the primary roles of the TN were to encourage the patient and caregiver to assert a more active role during care transitions, to provide continuity across settings, and to ensure that the patient’s needs were being met irrespective of the care setting. A personal health record (PHR) was used as a tool to engage patients in self-care. When the intervention targeted the professionals, the nurse developed an individualized discharge plan with the patient, health professionals, family, and caregivers [19, 22]. A summary of the discharge plan was distributed to the patient, primary care physician, and other health care team members who would care for the patient at home. When both professionals and the patient were targeted by the intervention, the development of a discharge plan was accompanied by patient and caregiver education in order to increase their ability to manage unresolved health problems once at home [27]. The interventions began upon admission to hospital and were continued for 1 week [24], 2 weeks [19], 4 weeks [20, 27], or 6 months [22] after hospitalisation. In two studies, the TN was partnered with a physiotherapist or a pharmacist [22, 24]. The most frequent action carried out by the TN was related to medications. Some data do indeed suggest that medication reconciliation should be a part of interventions aiming to reduce the risk of rehospitalisation [28].

Our care transition program targets health care professionals in and out of hospital and acts as an interface between them to provide patient-centred care. Coordination tools for these personnel have therefore been created. The TN also acts as a liaison between the professionals and the patient: she explains to the patient how his care is to be organised and assumes the role of alerting other health care professionals about the patient's clinical status. The profile of the TN corresponds to that found in the studies cited above, with experience in geriatrics preferred, even if the status of advanced practice nurse does not exist in France. Nurses who are independent with respect to the participating units were recruited in order to ensure that the TNs are fully committed to the project and that their work does not deviate toward the routine tasks of the units. This means that each unit must take the necessary steps to quickly integrate the TN.

Finally, the duration of follow-up at home has been limited to 4 weeks, a period during which hospital readmissions can be linked to health care insufficiencies during hospitalisation and at the time of discharge [5].

Our multicentre experimental study should make it possible to provide the scientific community with answers regarding the specific effect of a bridging intervention involving a designated transition professional who intervenes before and after discharge from hospital, in a real life context and with an assessment of medico-economic impact.

## Abbreviation

TN: Transition nurse
